# Endoscopic examination of labial fusion in a postmenopausal woman: a case report

**DOI:** 10.1186/s13256-018-1568-4

**Published:** 2018-02-02

**Authors:** Yusaku Kumagai, Masafumi Toyoshima, Kei Kudo, Minoru Ohsawa, Hitoshi Niikura, Nobuo Yaegashi

**Affiliations:** Department of Obstetrics and Gynecology, Tohoku University Hospital, 1-1, Seiryo, Aoba, Sendai, Miyagi 980-8574 Japan

**Keywords:** Dysuria, Endoscopic examination, Labial fusion, Postmenopausal woman

## Abstract

**Background:**

Labial fusion is defined as adhesions of the labia minora or majora. Labial fusion may cause urinary retention. Surgical treatment based on an accurate anatomic assessment may be needed, but the usefulness of endoscopic examination for this disease has not been reported.

**Case presentation:**

A 76-year-old Japanese woman undergoing chemoradiation treatment for esophageal cancer was referred to our department for evaluation of high accumulation in the vagina on a positron emission tomography scan. On physical examination, her labia were noted to be extensively fused with a pinhole opening at the midline. Endoscopic examination revealed that her vagina was filled with urine and there were no abnormalities in her urethral meatus and cervix. The adhesions were separated under anesthesia and there has been no recurrence during follow-up.

**Conclusions:**

We present a case of a postmenopausal patient with labial fusion who underwent successful surgical management. An endoscopic examination enabled us to determine the precise anatomic position and adopt a safe surgical procedure.

**Electronic supplementary material:**

The online version of this article (https://doi.org/10.1186/s13256-018-1568-4) contains supplementary material, which is available to authorized users.

## Background

Labial fusion is a benign genital disorder which is most commonly described in prepubertal girls, and less often reported in postmenopausal women [1, 2]. The etiology of labial fusion in the reproductive or postmenopausal age group is unknown. Labial fusion can be caused by infection [3, 4], trauma to the genitalia [2], or chronic inflammation resulting in low serum estrogen levels [5]. Labial fusion is diagnosed by visual inspection, and the clinical complications associated with labial fusion are usually minor. However, urinary tract infection or hydronephrosis can result from disturbances in urination [6, 7]. Surgical treatment is needed if a patient complains of urination disorders or the degree of labial fusion is severe. These adhesions are usually superficial, but sometimes involve the clitoris, thus making it difficult to distinguish the precise anatomic location. Although an endoscopic examination is frequently performed in the diagnosis and treatment of lower and upper urinary tract disease, the use of cystoscopy in patients with labial fusion and urinary retention has not been reported. We present a case of total labial adhesions in a postmenopausal woman who underwent an endoscopic examination.

## Case presentation

**Fig. 1:**
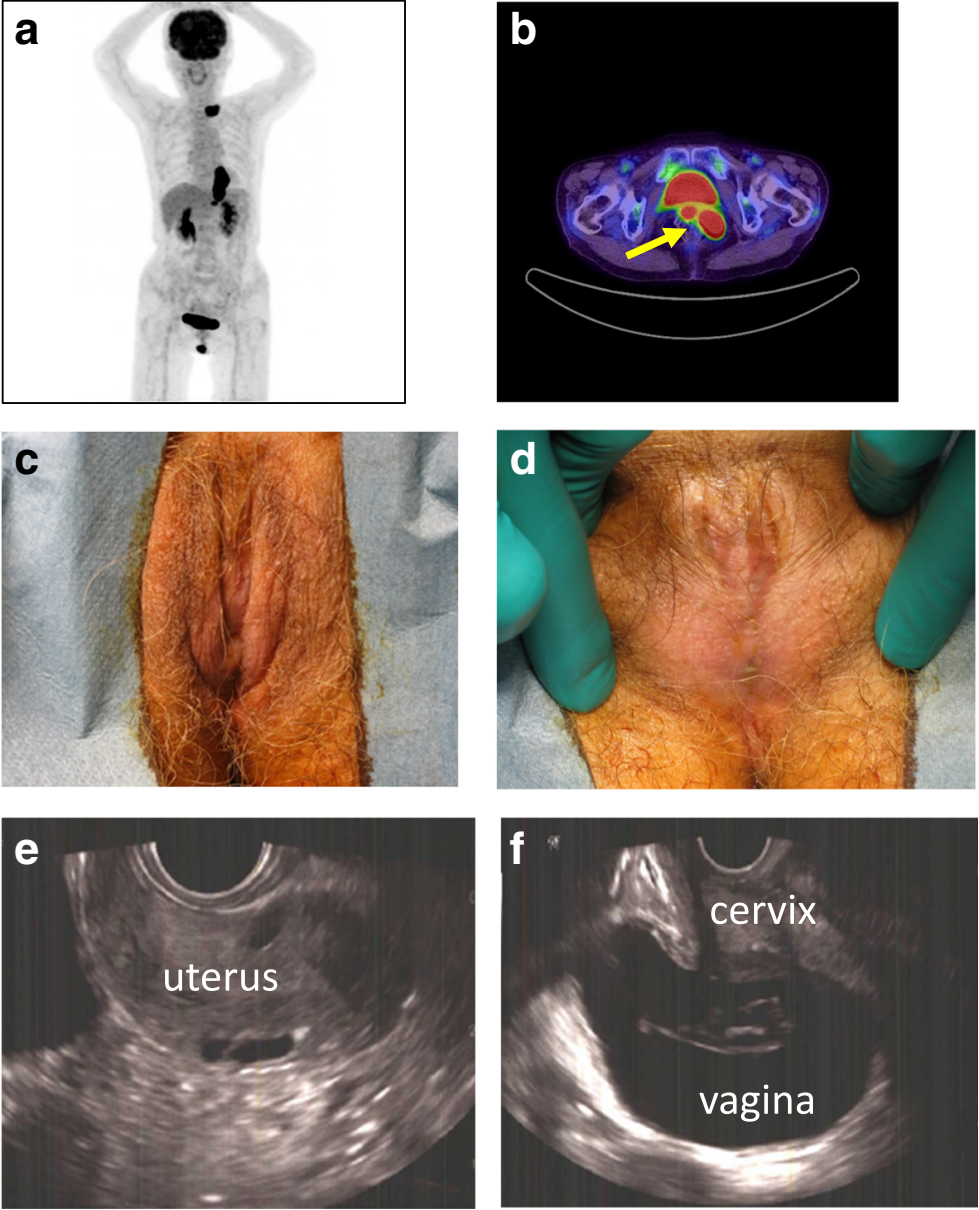
Examination findings at Out-patient Department. **a** Several hot spots were detected within the esophagus SUVMax 14.2), the left supraclavicular lymph node SUVMax 8.2), and the lesser curvature of the stomach SUVMax 5.5) in positron emission tomography scan. **b** A high accumulation, SUVMax 39.1, was found in the vagina and the cervix (*arrow*). **c** Inspection of the vulva. **d** The labia majora is completely fused and there is a pinhole opening in the midline. The vaginal introitus and urethral meatus are obliterated. **e** A transrectal ultrasound revealed the uterus was retroflexed and the endometrium was thin. **f** The vagina was dilated with fluid retention and a normal cervix was visualized

A 76-year-old Japanese woman, who received palliative chemoradiation treatment for stage IV esophageal cancer, was referred to our Gynecology Out-patient Department for evaluation of high accumulation in the vagina, maximum standardized uptake value (SUVMax) of 39.1, on a positron emission tomography (PET) scan (Fig. 1a, b). She had no complaints of difficulty with urination, except that it took a long time to urinate. She denied pain while urinating and urinary frequency. A urine analysis did not reveal hematuria or bacteriuria. She had two vaginal deliveries and no history of major trauma in external genitalia at birth. She denied past history of pelvic trauma or infectious disease in the external genitalia. She was menopausal at 40 years of age, and had never undergone a Papanicolaou (Pap) smear for cervical cancer screening.

**Fig. 2:**
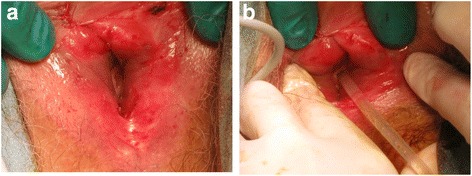
**a** Postoperative view of the vaginal introitus. **b** A urethral catheter was inserted in the meatus urethra


Inspection of her external genitalia showed that her clitoris was normal in size, and the labia majora and anus were visualized (Fig. 1c); however, the urethral meatus and vagina orifice were not seen on careful inspection of her vulva (Fig. 1d). On palpation of her external genitalia, there was a pinhole opening at the midpoint between her clitoris and anus. A transrectal ultrasound revealed an atrophic uterus (Fig. 1e) and large amount of fluid retention in her vagina (Fig. 1f). A cystoscopic examination was performed in an effort to define the anatomic structures in the closed vaginal cavity before an invasive procedure was undertaken (Additional file 1: Video 1). The pinhole was dilated and a fiber was inserted into the hole. The tip of the fiber reached her bladder, and we confirmed that the urethra, cervix, and urethral meatus within her vagina were filled with urine. Thus, surgical intervention was recommended. Under topical anesthesia, the labial adhesions were bluntly and sharply dissected (Fig. 2a, b). A Pap smear showed no cervical abnormalities. We gave our patient local hygiene recommendations and applied an estrogen ointment to prevent reformation of adhesions. She is currently healthy and has had no recurrence 3 months after surgery.

**Additional file 1: Video 1.** A cystoscopic examination revealed the vagina was filled with urine and there were no abnormalities in the cervix and urethral meatus. (MP4 5212 kb)


## Discussion

Labial adhesions are defined as partial or complete adherence of the labia minora or majora. A low serum estrogen level is the basic cause of labial adhesions [8]. Physiologic hypoestrogenism, together with chronic inflammation in the vulvar skin and mucosa, leads to labial adhesions with subsequent partial or total obstruction of the vagina and/or the urethra. Because our patient had received radiation therapy in our hospital, decreased general activity and lack of sexual intercourse were the major causative factors leading to labial fusion.

An endoscope is widely used to directly visualize various organs inside the human body. Because endoscopes are small and flexible and feature a high-resolution camera which enables a clear view, the application of endoscopy has increased. In the current case, our patient had a pinhole opening in the adherent labia and her vagina was filled with urine. A cystoscope was inserted through this pinhole and her bladder, urethra, and cervix were visualized (Additional file 1: Video 1). In fact, an endoscopic examination made it possible to confirm the precise anatomic structures and relationships beyond the adhesions.

The treatments which are available for symptomatic patients and those with severe adhesions include non-surgical and surgical methods. Topical estrogen and betamethasone treatment with good vulvar hygiene is generally selected as first-line treatment for prepubertal girls; the success rate is between 50 and 88% [9]. In contrast, estrogen therapy is not always successful for adult patients [9]. Surgical separation should be considered in cases that are not responsive to conservative management or when immediate treatment is required. Seehusen and Earwood reviewed ten cases of postpartum labial adhesions and reported that surgical dissection was definitive therapy in every reported case [2]. Postoperative estrogen cream should help prevent recurrences and repeat surgery [10]. The significance of this case is that it is the first report of labial adhesions in which an endoscopic examination facilitated safe surgical treatment.

## Conclusions

In this report, we present a case of a postmenopausal patient with esophageal cancer who had an abnormal finding involving the vagina on a PET scan. The diagnosis of labial fusion can be made by inspection, but endoscopic examination was shown to be invaluable to determine the precise anatomic structures and relationships, thus enabling us to perform a safe surgical procedure.
